# Follow-up of a healthy lifestyle education program (the EdAl study): four years after cessation of randomized controlled trial intervention

**DOI:** 10.1186/s12889-017-5006-0

**Published:** 2018-01-05

**Authors:** Elisabet Llauradó, Lucia Tarro, David Moriña, Magaly Aceves-Martins, Montse Giralt, Rosa Solà

**Affiliations:** 1 Facultat de Medicina i Ciències de la Salut, Health Education and Promotion, Functional Nutrition, Oxidation and Cardiovascular Disease Research Group, Medicine and Surgery Department, Universitat Rovira i Virgili, C/Sant Llorenç, 21, 43204 Reus, PC Spain; 2 Unit of Infections and Cancer (UNIC – I&I), Cancer Epidemiology Research Program (CERP), Catalan Institute of Oncology, (ICO)-IDIBELL, L’Hospitalet de Llobregat, Barcelona, Spain; 3 Unit of Farmacobiology, Functional Nutrition, Oxidation and Cardiovascular Disease Research Group, Medicine and Surgery Department, Universitat Rovira i Virgili, C/Sant Llorenç, 21, 43204 Reus, PC Spain; 4 Unit of Lipids and Arteriosclerosis Research, CIBERDEM, Hospital Universitari Sant Joan, IISPV, Universitat Rovira i Virgili, Technological Centre of Nutrition and Health (CTNS), Functional Nutrition, Oxidation and Cardiovascular Disease Research Group, Medicine and Surgery Department, Universitat Rovira i Virgili, C/Sant Llorenç, 21, 43204 Reus, PC Spain

**Keywords:** Follow-up, School-based intervention, Obesity, Lifestyles, Adolescents

## Abstract

**Background:**

An important challenge of school-based childhood obesity (OB) intervention programs is understanding the maintenance of the effects after cessation of the intervention to overcome the limitations of follow-up studies. The aim of this study is to verify the sustainability of the benefits achieved at a 4-year follow-up of the post-*Educació en* Alimentació (EDAl) program intervention cessation by assessing the OB-related outcomes and lifestyles of 13- to 15-year-old adolescents.

**Methods:**

This paper describes a 4-year follow-up study after the cessation of a school-based randomized controlled intervention in adolescents (*n* = 349, intervention; *n* = 154, control) with baseline and 4-year follow-up data from high schools in Reus (intervention group), Salou, Cambrils and Vila-seca (control group). The outcomes are body mass index (BMI), BMI z-score, and OB prevalence according to the World Health Organization and International Obesity Task Force criteria and lifestyle data (obtained from questionnaires).

**Results:**

Compared with the control girls, the intervention girls showed reduced BMI z-scores (−0.33 units, *p* < 0.01) from baseline (2007) to the 4-year follow-up post-intervention (2014). Compared with the control boys, the intervention boys showed reduced OB prevalence (−7.7%; *p* = 0.02). Compared with the control boys, more boys in the intervention group (19% increase; *p* = 0.059) showed ≥4 h/week after-school physical activity (PA). A decrease in the consumption of dairy products, fruits and fish was observed in both groups.

**Conclusions:**

At the 4-year post-intervention follow-up of the EdAl program, compared with the control groups, girls had lower BMI z-scores and boys had lower OB prevalence from the intervention. The encouragement in after-school PA was long-lasting and maintained after the cessation of the intervention, whereas healthy food habits must be further reinforced in adolescents.

**Trial registration:**

ISRCTN29247645.

**Electronic supplementary material:**

The online version of this article (10.1186/s12889-017-5006-0) contains supplementary material, which is available to authorized users.

## Background

Childhood obesity (OB) is a public health challenge [1]. The most recent data show that approximately one in five children in the Organisation for Economic Co-operation and Development (OECD) area are overweight (OW) or obese [2]. In Spain, childhood OW and OB trends have continuously grown [3]. However, the childhood OW and OB classification from the OECD countries [4] showed higher values in Spanish children than the OECD mean data. In Spain, the OB prevalence was 6.7% in the adolescent population (14–17 y), as measured by the International Obesity Task Force (IOTF) [5].

To discover effective interventions that reduce OB over the long-term after the cessation of an intervention is challenging, and scarce information is available for this purpose [6]. A Cochrane review of interventions for preventing OB in children and adolescents emphasized that education may make it possible to prevent OB and that the long-term effects of an intervention should be assessed [6].

From the school-based programs that have been identified, few have been evaluated post-intervention for their follow-up effectiveness because such evaluations are difficult due to economic concerns and high-rates of participant dropout that induce a low-attrition rate and logistical troubles [7]. There are several examples of post-intervention follow-up effectiveness studies, such as the Cretan Health and Nutrition Education Program [8, 9] at 4-y follow-up post-intervention, the Child and Adolescent Trial for Cardiovascular Health (CATCH) cohort at 3-y follow-up post-intervention, [10, 11] the CHristchurch Obesity Prevention Programme in Schools (CHOPPS) study, [12] the A Pilot Programme for Lifestyle and Exercise (APPLE) project [13] at 2-year follow-up post-intervention, and the Intervention in Eating Habits and Physical Activity in Schoolchildren. The AVall study at 2- and 4-year follow-up post-intervention [14, 15] demonstrated long-lasting effects in physical activity practice or OB prevalence post-cessation of the intervention.

We have previously shown that the effects of the primary school-based Educació en Alimentació (EdAl) program that was applied in a Spanish area (24 schools, *n* = 1222 in the intervention group and 14 schools and *n* = 717 in the control group) resulted in a successful reduction in the prevalence of childhood OB in boys by 4.39%. Compared with the control boys, the boys in the intervention group showed a BMI z-score reduction of −0.24 units; the effectiveness of an intervention is considered to be a BMI z-score reduction of more than −0.15 units of the [16]. Notably, compared with the controls, 5.1% more pupils in the intervention group engaged in >5 h/week of after-school physical activity (PA) after the 28 months of intervention, which ended in 2010 [17]. Furthermore, in 2012, at the 2-year follow-up post-intervention, these effectiveness patterns had been maintained: compared with the control groups, both genders showed lower BMI z-scores, lower OB prevalence, and increased after-school PA [18]. To overcome the limitations of follow-up studies, the aim of the present study is to verify the sustainability of the benefits achieved at 4-year follow-up post-intervention by assessing the OB-related outcomes and lifestyles of 13- to 15-year-old adolescents in 2014, 4 years after the cessation of EdAl intervention participation.

## Methods

### Aim, design and setting of the study

The aim of the present study is to verify the sustainability of the benefits that were achieved at 4-year follow-up post-EdAl intervention by assessing the OB-related outcomes and lifestyles in 13- to 15-year-old adolescents in 2014, 4 years after the cessation of EdAl intervention participation.

The protocol, rationale, design and procedures of the EdAl program (trial registration number ISRCTN29247645), as well as the results obtained at the conclusion of the study and at 2-year follow-up, have been published [17, 18, 19]. The current study, which was conducted at the 4-year follow-up post-intervention in 2014, was approved by the clinical research ethical committee of the *Hospital Universitari Sant Joan de Reus, Universitat Rovira i Virgili* (Catalan ethical committee registry #20; ref.: 12–03-29/3proj2).

The protocol conformed to the Helsinki Declaration and Good Clinical Practice guidelines of the International Conference of Harmonization (ICH GCP). The data that were collected on the adolescents, who provided written informed consent (signed by the parents or guardians) prior to their participation in the follow-up study, were analyzed. The 4-year follow-up post-intervention of the EdAl program was an observational study and is described according to the STROBE Statement [20].

Briefly, the EdAl program consisted of 12 educational intervention activities [16, 18] that focused on 8 lifestyle topics selected based on the scientific evidence to improve nutritional food selection, healthy habits such as teeth brushing and hand washing, the overall adoption of behaviors that encourage PA (i.e., walking to school and playground games) and the avoidance of sedentary behavior. These intervention activities were based on 12 activities (1 h/activity/session) conducted 4 per year every 15 days in the third trimester of a Spanish academic course (April to June) over 15 weeks per academic year [21]. The design, standardization and implementation were made by university students who acted as health promoting agents (HPAs) to 7- and 8-year-old children at primary schools over 28 months during 3 academic years that ended in 2010. All activities were described in a lesson planning format, a tool that is usually used by primary school teachers. All the activities had the same following format: 5–10 min of funny theory about nutritional characteristics or health benefits; 15 min of play based on the theory of this activity (for example, memory cards); 30 min of experimental activity (children played and tasted the food that related to the activity); and 5–10 min of discussion and to answer questions. The university students used the service-learning method to develop activities and practices that were geared toward children, and the HPAs’ reflections related their service to their academic work [21–23]. Moreover, each intervention activity had an educational message that related to one of the following eight lifestyle topics: (First year) (1) to improve toward a healthy lifestyle; (2) to encourage the intake of healthy drinks (and the avoidance of unhealthy carbonated/sweetened beverages); (3) to increase the consumption of vegetables and legumes; (4) to decrease the consumption of candies and pastries while increasing the intake of fresh fruits and nuts; (Second year) (5) to improve healthy habits within a set timetable (i.e., home-cooked meals, teeth brushing, and hand washing) and PA participation; (6) to increase fruit intake; (7) to improve dairy product consumption; and (8) to increase fish consumption. In the Third year, these eight lifestyle topics were reinforced.

Furthermore, the parents were involved in these activities with their children. For each activity, the children took home some recommendations on healthy lifestyles and shared with their parents the information of the activity that was developed at school.

Optionally, depending on the schools and parents, the same educational activities that the children participated in were offered to their parents. In this way, the parents and children interacted with the same healthy nutrition and lifestyle activities.

The fidelity of the schools was great. All of the participating schools were enthusiastic with all the activities of the EdAl program. Even the schools in the control group were pleased with the program, and the university offered to implement the intervention in the control group schools when the long-term effectiveness of the study is demonstrated.

### Re-recruiting of adolescents

The children participants of the EdAl study at the 2-year follow-up post-cessation changed from primary school to high school. The local authorities of the participants’ towns provided us the name of the current high school of these participants to contact their parents/legal guardian and themselves. Then, researchers contacted the directors of these high schools and the parents of the participants to request permission to repeat the anthropometric measures and distribute the lifestyles questionnaire. Next, researchers assessed the EdAl participants at 2-year follow-up post-intervention [18] and repeated the assessment at 4-year follow-up post-intervention.

**Fig. 1:**
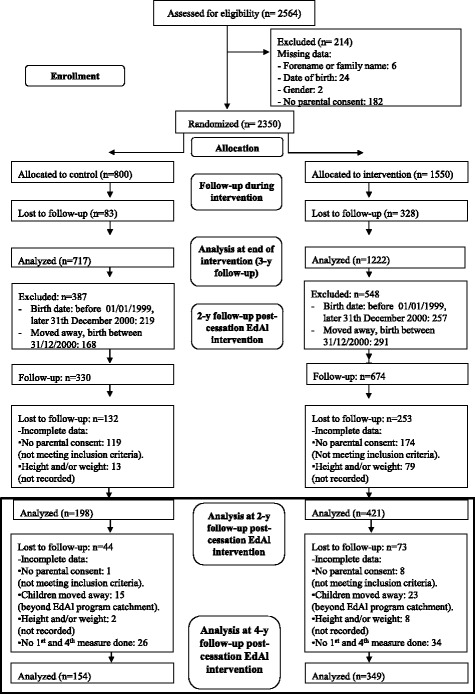
Flow diagram of the participants throughout the study. The 4-year follow-up post-intervention measurements were conducted over the 2014 academic year in Reus (intervention group) and in Cambrils, Salou and Vila-seca (control group)

If any adolescent changed to another high school and did not further participate in the EdAl study, these adolescents were considered to be dropouts. Another dropout reason at 2-year follow-up post-intervention was done by the authors’ criteria of excluded the participants birth before 01/01/1999 and after 31/12/2000 [18], due to this participants did not present the inclusion criteria data completed (Fig. 1).

### Outcomes

Weight and height were obtained as described in the protocol [19]. The primary outcome was OB prevalence measured as BMI according to IOTF [24] and World Health Organization (WHO) criteria [25]. Secondary outcomes were OB-related outcomes such as BMI, BMI z-score, waist circumference, hip circumference, incidence and remission of excess weight (i.e., the participant’s change from OB status to OW or normal weight). The BMI z-score was analyzed according to the WHO Global InfoBase that defines children with a BMI z-score > 2 as OB [25]. Additionally, dietary habits (such as breakfast and its composition of dairy products, cereal and pastries, the consumption of fruit, vegetables, second dairy products, fish, legumes, candy, pasta, rice, fast food and olive oil and cooking at home) were analyzed by using the enKid questionnaire [26]. These dietary habits (such as the composition of breakfast before leaving home and the snack at midmorning), after-school PA in hours/week and sedentary lifestyles (television and/or video games in hours/day) were recorded using the AVall questionnaire [27]. Both questionnaires were completed at baseline (2007–2008) and at the 4-year follow-up post-intervention (2014).

### Statistical analyses

The descriptive data are presented as the means or percentages and 95% confidence intervals (95% CIs) or ± standard deviations (±SDs) for the variables that followed normal distributions. General linear models (GLMs) were used to analyze the differences between the continuous values for the intervention and control groups in relation to the prevalence of OB. The anthropometric data were analyzed using an ANOVA adjusted for age. Repeated measures GLMs were used to analyze the trend in the BMI z-scores between baseline and the 4-year follow-up post-intervention. McNemar’s test was used to calculate the differences among the changes from the baseline to the 4-year follow-up post-intervention in both groups.

**Fig. 2:**
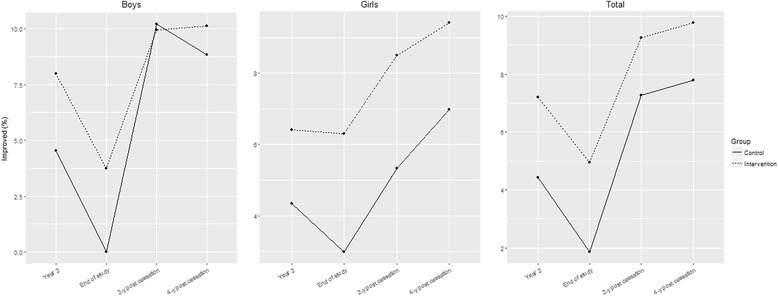
Evolution of the proportion of the children who improved in the BMI category since the previous time point

The primary analyses were performed with the modified intention-to-treat (mITT) population, that is, the subjects with at least baseline and 4-year follow-up post-intervention measurements of weight and height. The analysis did not use any imputation missing method, with the assumption that any missing data were random. The statistical significance was set at *p* ≤ 0.05. The data were analyzed by using SPSS software (version 22) and R version 3.3.3 [28]. Fig. 2 was generated by using the R package ggplot2 [29].

## Results

At the 4-year follow-up post-intervention, 349 of 966 (36.12%) adolescents in the intervention group and 154 of 413 (37.28%) adolescents in the control group were included in the analysis (Fig. 1). The observed dropouts were not more prevalent among either the OW or OB adolescents.

At the 4-year follow-up post-intervention, the mean (±SD) age was 15.6 ± 0.53 years in the intervention group and 14.9 ± 0.36 years in the control group (*p* < 0.01) and was not different between the genders.

An analysis of the anthropometric characteristics of the adolescents at the 4-year follow-up post-intervention showed that the BMI and waist circumference were greater in the boys in the intervention group than the boys in the control group. In contrast, the control girls presented with 2.45 kg more of fat mass compared with the intervention girls (15.47 kg vs. 13.02 kg; *p* = 0.008) (Table 1).

**Table 1:** Anthropometric characteristics of the students at 4-year follow-up post-intervention in the intervention and control groups

	Intervention group (*n* = 349)			Control group (*n* = 154)			*p*-value intervention vs. control		
	Boys	Girls	Total	Boys	Girls	Total	Boys	Girls	Total
Weight; kg^a,b^	57.87 (56.10 to 59.65)	52.44 (50.94 to 53.93)	55.17 (53.99 to 56.35)	55.24 (52.12 to 58.36)	54.44 (52.22 to 56.67)	54.89 (52.99 to 56.79)	0.178	0.169	0.819
Height; cm^a,b^	167.6 (166.4 to 168.8)	160.9 (159.9 to 161.9)	164.3 (163.4 to 165.1)	167.7 (165.6 to 169.9)	161.5 (160.1 to 163.0)	164.4 (163.0 to 165.8)	0.944	0.496	0.911
BMI^1^; kg/m^a,e^	20.50 (20.05 to 20.95)	20.31 (19.86 to 20.76)	20.41 (20.09 to 20.72)	19.44 (18.59 to 20.30)	20.60 (19.90 to 21.30)	20.09 (19.54 to 20.64)	**0.020**	0.475	0.294
BMI z-score^a,e^	−0.10 (−0.27 to 0.08)	−0.21 (−0.36 to −0.06)	−0.15 (−0.27 to −0.04)	−0.37 (−0.66 to −0.09)	−0.01 (−0.23 to 0.21)	−0.17 (−0.35 to 0.00)	0.100	0.139	0.846
Fat mass; kg^a,d,e^	8.24 (7.55 to 8.93)	13.02 (12.12 to 13.92)	10.58 (9.96 to 11.19)	7.92 (6.48 to 9.35)	15.47 (13.66 to 17.28)	12.11 (10.79 to 13.44)	0.653	**0.008**	**0.018**
Lean mass; kg^a,d^	49.65 (48.35 to 50.96)	39.82 (39.09 to 40.56)	44.78 (43.88 to 45.66)	47.87 (45.57 to 50.17)	40.97 (39.87 to 42.07)	44.20 (42.75 to 45.66)	0.217	0.111	0.538
Waist circumference; cm^a,b^	74.15 (72.88 to 75.43)	69.88 (68.73 to 71.02)	72.06 (71.17 to 72.94)	71.57 (69.36 to 73.78)	71.15 (68.98 to 73.31)	71.33 (69.80 to 72.87)	**0.043**	0.261	0.401
Hip circumference; cm^a^	84.01 (82.65 to 85.37)	82.92 (81.72 to 84.12)	83.46 (82.56 to 84.37)	81.76 (79.12 to 84.41)	85.09 (83.12 to 87.07)	83.66 (82.04 to 85.27)	0.162	0.082	0.848

The 4-year follow-up post-intervention measurements were conducted over the 2014 academic year in Reus (intervention group) and in Cambrils, Salou and Vila-seca (control group)

Bold text indicates significant *p*-values

^a^The results are expressed as the means (95% CIs)

^b^Weight, height, lean mass and hip circumference; values adjusted by age with an analysis of covariance (ANCOVA)

^c^Body mass index (BMI) is calculated as weight (kg) divided by height in square meters

^d^Fat and lean mass are calculated by using a standard beam balance (Tanita TBF-300 Body Composition Analyzer)

^e^BMI, BMI z-score and fat mass, waist circumference, not adjusted by age, are calculated by using an analysis of variance (ANOVA)

At 4-year follow-up post-intervention, the BMI z-scores in the intervention groups were reduced by −0.58 units, whereas in the control groups, it was reduced by −0.41 units, with a significant difference of −0.17 units (*p* = 0.003). By gender, in girls, compared with a BMI z-score reduction of −0.32 in the control group, the BMI z-score was reduced by −0.65 in the intervention group, with a significant difference of −0.33 units (*p* = 0.003) (Table 2). In contrast, there was not a significant difference in the BMI z-score in boys (Table 2).

**Table 2:** BMI z-score at baseline and 4-year follow-up post-intervention in the intervention and control groups

	Baseline^c^	4-year follow-up post-intervention	Change baseline^c^ to 4-year follow-up post-intervention	Baseline^c^ vs. 4-year follow-up post-intervention	Intervention vs. control
	Mean (95% CI)	Mean (95% CI)	Mean (95% CI)	*p*-value^b^	*p*-value^d^
BMI Z-score^a^					
Intervention					
Boys	0.57 (0.40 to 0.74)	−0.09 (−0.25 to 0.08)	−0.67 (−0.80 to −0.54)	**<0.001** ^e^	0.282
Girls	0.44 (0.26 to 0.61)	−0.23 (−0.39 to −0.07)	−0.65 (−0.78 to −0.51)	**<0.001** ^e^	**0.003**
Total	0.51 (0.38 to 0.63)	−0.15 (−0.27 to −0.04)	−0.58 (−0.75 to −0.56)	**<0.001** ^e^	**0.003**
Control					
Boys	0.16 (−0.12 to 0.44)	−0.37 (−0.66 to −0.09)	−0.54 (−0.72 to −0.35)	**<0.001** ^e^	
Girls	0.30 (0.08 to 0.53)	−0.01 (−0.23 to 0.21)	−0.32 (−0.45 to −0.18)	**<0.001** ^e^	
Total	0.24 (0.06 to 0.42)	−0.17 (−0.35 to 0.00)	−0.41 (−0.53 to −0.30)	**<0.001** ^e^	

*BMI* body mass index, *CI* confidence interval, *WHO* World Health Organization

Bold text indicates significant *p*-values

The 4-year follow-up post-intervention measurements were conducted over the 2014 academic year in Reus (intervention group) and in Cambrils, Salou and Vila-seca (control group)

^a^The BMI z-score was calculated from the WHO “Growth reference 5–19 years” tables

^b^General Linear Mixed Models of repeated measures

^c^baseline (2007–2008)

^d^Fisher’s exact test

^e^repeated measures General Linear Mixed Models

Although no statistically significant differences were found between the control and intervention groups on OB and OW prevalence, which is probably due to a lack of power given the categorical nature of the variable, the intragroup evolution is clearly different. At the 4-year follow-up post-intervention, compared with the baseline prevalence, the OB prevalence by the WHO criteria was reduced by −11.8% in the boys in the intervention group and by −4.1% in the boys in the control group, with a difference of −7.7% (*p* = 0.019) in favor of the intervention group (Table 3). Conversely, at the 4-year follow-up post-intervention, the OW prevalence (according to the IOTF criteria) decreased in the girls in the intervention group (from 17.5% to 6.4%; *p* = 0.002) and in the girls in the control group (from 20.9% to 10.5%; *p* = 0.022) (Table 3). Improvement over time was also analyzed by tracking the changes in the BMI category over time through a mixed logistic model of repeated measures, without the ability to detect the differences between the groups for boys (*p* = 0.449), girls (*p* = 0.523) or overall (*p* = 0.291). The evolution of the proportion of the adolescents who improved after the study is shown in Fig. 2.

**Table 3:** Baseline and 4-year follow-up post-intervention measurements of BMI categorized as OW and OB in the intervention and control groups

		Baseline, *n* (%)	4-year follow-up post-intervention, *n* (%)	*p*-value Baseline to 4-year follow-up post-intervention^a^	*p*-value Intervention vs. Control^b^
WHO Criteria^c^					
OW					
Intervention	Boys	39 (21.9)	31 (17.4)	0.322	0.314
	Girls	38 (22.2)	12 (7.0)	**<0.001**	0.602
	Total	77 (22.1)	43 (12.3)	**<0.001**	0.238
Control	Boys	8 (11.8)	6 (8.8)	0.774	
	Girls	18 (20.9)	12 (14.0)	0.286	
	Total	26 (16.9)	18 (11.7)	0.229	
OB					
Intervention	Boys	24 (13.5)	3 (1.7)	**<0.001**	**0.019**
	Girls	11 (6.4)	4 (2.3)	**0.039**	0.560
	Total	35 (10.0)	7 (2.0)	**<0.001**	0.205
Control	Boys	5 (7.4)	3 (4.4)	0.500	
	Girls	6 (7.0)	2 (2.3)	0.289	
	Total	11 (7.1)	5 (3.2)	0.109	
IOTF Criteria^d^					
OW					
Intervention	Boys	39 (21.9)	30 (16.9)	0.163	0.238
	Girls	30 (17.5)	11 (6.4)	**0.002**	0.581
	Total	69 (19.8)	41 (11.7)	**0.001**	0.244
Control	Boys	6 (8.8)	3 (4.4)	0.375	
	Girls	18 (20.9)	9 (10.5)	**0.022**	
	Total	24 (15.6)	5 (3.2)	**0.008**	
OB					
Intervention	Boys	4 (2.2)	1 (0.6)	0.375	0.578
	Girls	8 (4.7)	4 (2.3)	0.289	0.430
	Total	12 (3.4)	5 (1.4)	0.092	0.186
Control	Boys	3 (4.4)	3 (4.4)	1.000	
	Girls	1 (1.2)	2 (2.3)	1.000	
	Total	4 (2.6)	5 (3.2)	1.000	

*BMI* body mass index, *IOTF* International Obesity Task Force, *OW* overweight, *OB* obesity, *WHO* World Health Organization

Bold text indicates significant values

The 4-year follow-up post-intervention measurements were conducted over the 2014 academic year in Reus (intervention group) and in Cambrils, Salou and Vila-seca (control group)

^a^Fisher’s exact test

^b^McNemar’s test

^c^The WHO criteria cutoff points (2007) were used for the BMI classification

^d^The IOTF criteria cutoff points (Cole, 2000) were used for the BMI classification

At the 4-year follow-up post-intervention, the percentage of the adolescents in the intervention group who spent ≤2 h/day watching TV and/or playing video games significantly decreased by 91.2% (*n* = 237) to 73.3% (*n* = 231) (*p* = 0.001), but compared with the control group, no such differences were observed (Table 4).

**Table 4:** Physical and leisure activities assessed at baseline and 4-year follow-up post-intervention in the intervention and control groups

		Intervention group			Control group			
		Baseline, *n* (%)	4-year follow-up post-intervention, n (%)	*p*-value baseline to follow-up^b^	Baseline, *n* (%)	4-year follow-up post-intervention, n (%)	*p*-value baseline to follow-up^b^	*p*-value intervention vs. control changes^c^
TV and/or video games, h/day								
≤ 2 h/day	Boys	120 (90.9)	113 (72.0)	0.002	34 (81.0)	47 (78.3)	0.754	0.084
	Girls	117 (91.4)	118 (74.7)	0.002	58 (85.3)	49 (73.1)	0.109	0.919
	Total	237 (91.2)	231 (73.3)	<0.001	92 (83.6)	96 (75.6)	0.115	0.268
After-school physical activity, h/week								
≥ 4 h/week	Boys	41 (31.1)	92 (57.9)	<0.001	10 (24.4)	22 (36.7)	0.388	0.111
	Girls	27 (21.6)	59 (37.6)	0.004	13 (19.7)	22 (31.9)	**0.049**	0.852
	Total	68 (26.5)	151 (47.8)	<0.001	23 (21.5)	44 (34.1)	**0.024**	0.174

Bold text indicates significant p-values

The 4-year follow-up post-intervention measurements were conducted over the 2014 academic year in Reus (intervention group) and in Cambrils, Salou and Vila-seca (control group)

^a^Physical activity and the number of TV hours were analyzed at baseline and at 4-year follow-up post-intervention

^b^McNemar’s test

^c^Fisher’s exact test of differences between the intervention and control changes

Furthermore, we observed an increase in the number of the participants with ≥4 h/week of after-school PA at the 4-year follow-up post-intervention. This increase was 26.8% (*p* < 0.001) in the boys and 16% (*p* = 0.004) in the girls in the intervention group, whereas this increase was 12.3% (*p* = 0.388) in the boys and 12.2% (*p* = 0.049) in the girls in the control group. Compared with the boys in the control group, more boys in the intervention group (an increase of 19%; *p* = 0.059) performed ≥4 h/week of after-school PA, whereas in girls, the difference between the two groups was not significant (Table 4).

Based on the 15 food items in the Krece Plus questionnaire, the 4-year follow-up post-intervention showed deteriorating food behaviors that related to the consumption of dairy products, fruit and fish in both the intervention and control groups, primarily in the girls (Additional file 1). However, considering the dietary items on the AVall questionnaire, we observed an increase of 11.1% (*p* = 0.039) in sandwich consumption for breakfast before leaving home in the control group compared with the intervention group. In the intervention group, we observed a decrease in the midmorning breakfast consumption of pastries (*p* = 0.029), juice and soft drinks compared with the control group (*p* = 0.009) (Additional file 1).

## Discussion

This study reports the long-lasting effects of the EdAl school-based OB intervention program at the 4-year follow-up post-intervention. The results show an effective significant reduction of −0.33 units in the BMI z-score of the girls in the intervention group relative to the girls in the control group, as well as the effective significant reduction of −0.17 of the BMI z-score units in the participants overall. In addition, compared with the prevalence of OB boys in the control group, the findings show a reduction of −7.7% in the prevalence of OB in the boys in the intervention group.

In the present study, the BMI z-score reduction is effective considering the reductions of −0.15 units in the BMI z-scores between the pre- and post-intervention group changes relative to the control group [16]. The decrease in OB-related outcomes in the boys in the EdAl program began during implementation and continued after the cessation of the intervention, whereas the reduction in OB-related outcome effects in the girls were not observed until the 4-year follow-up post-intervention. Although the BMI z-score considers standard deviation units above or below the mean [30] that are determined according to the WHO population standard, OB prevalence reduction only reflects changes from OB to other weight statuses, such as OW and/or normal weight. These data suggest that the EdAl program was effective and long-lasting in the 4-year follow-up post-intervention with respect to OB-related outcomes. In the girls, the decreasing BMI z-score was nearer to the mean of the WHO population standard, while in the boys, the weight status decreased to a healthier category.

Furthermore, a measure of the beneficial effects of an intervention in terms of healthy lifestyle maintenance over the long term [31] is needed to serve as an indicator of the effectiveness of the intervention. According to our knowledge, there are seven follow-up studies that were performed post-intervention, including 3 studies that observed the effects at the 4-year follow-up post-intervention, [9, 15, 32, 33] such as the present study, which demonstrates the considerable difficulty in following the participants when the intervention ended.

For example, the AVall study, a 2-year school-based cluster randomized controlled trial (5- to 6-year-old children) that was implemented in Granollers (Spain), achieved a 3.6% reduction in the prevalence of OB in the intervention schoolchildren and a 0.5% increase in OB prevalence in the control schoolchildren at the 2-year follow-up post-intervention[14]. At the 4-year follow-up post-intervention, the BMI in the intervention group was reduced by 1.13 kg/m^2^ [15]. These findings demonstrate effective results at the 2-year follow-up post-intervention and slightly small improvements at the 4-year follow-up post-intervention. In contrast, the present study shows improvements in the BMI z-score and OB prevalence at the 4-year follow-up post-intervention, although the age of the target groups was different; in the AVall study, there were fewer young participants than in the EdAl study. Moreover, the Cretan study, a 6-year school-based cluster randomized controlled trial (1st grade school children) implemented in three counties in Greece demonstrated that the intervention group presented a lower increase in BMI at the 4-year follow-up post-intervention relative to the control group [8] In addition, the KOPS study, a 1-year school-based cluster randomized controlled trial (5–7-year-old children) that was implemented in Kiel (Germany) showed a sustainment in the incidence and remission of OW and no change in the OB-related outcomes at the 4-year follow-up post-intervention, with more OB presence in families with high socioeconomic status [32]. Conversely, the EdAl program collected no data related to family socioeconomic status, and this variable may be an OB confounder [34].

These 4-year follow-up post-intervention studies showed similarities with respect to beneficial changes in OB-related outcomes; however, comparing these studies is difficult because of the different OB-related measures that are used in each study. The data observed in the present work argue in favor of the measurement of various OB-related outcomes, including the BMI z-score of schoolchildren, to enable comparisons among different interventions.

A notable finding is that there is no consensus regarding the best way to identify OB in children and adolescents, which has contributed to some limitations because approaches differ with respect to BMI reference criteria, pubertal stages, and racial/ethnic differences [35]. A meta-analysis reported that the BMI criteria have high specificity but low sensitivity for identifying pediatric OB [35]. The BMI z-score is the optimal measure of annual adiposity change for elementary school-age children [36].

The present study showed an increase of PA at the 4-year follow-up post-intervention. Of 3 studies that reported the data of a 4-year follow-up post-intervention, only one of them provided data on PA variables. Thus, Cretan’s study [9] reported no changes of moderate-to-vigorous levels of PA in min/week in boys of the intervention group of students compared with the control group at 4-year follow-up post-intervention. Similarly, at 4-year follow-up post-intervention, the EdAl study demonstrated that, compared with the boys in the control group, the boys in the intervention group showed increased after-school PA in h/week. Although the moderate-to-vigorous levels of PA in the EdAl study was not assessed similar to Cretan study, both studies show only an increase in the PA of boys. Thus, future studies should identify strategies to motivate girls to be physically active.

In contrast, the CATCH study, a 3-year school-based randomized controlled trial (3rd-5th grade school children) that was implemented in the USA showed that the PA levels of intervention students declined after the cessation of the intervention, while the PA levels in the intervention group were higher than the PA levels of the control group [11]

At the 4-year follow-up post-intervention of the EdAl study, the food habits followed a similar pattern between the intervention and control groups and showed a reduction in the consumption of healthy foods such as dairy products, fruit and fish. This result suggests that the improvements in food-related habits that were established during the program are lost over time. This finding leads us to recommend that healthy dietary habits should be encouraged at the 2-year follow-up post-intervention to maintain healthy food intake. To our knowledge, long-term follow-up studies after the conclusion of school-based interventions for the prevention of OB have included data on OB-related outcomes and PA practice, but the subjects’ food habits have not been reported in the long-term post-intervention follow-ups. [10, 15]. This omission supports the need for further research in this field.

Some factors that may contribute to a publication bias due to the low number of follow-up publications on school-based interventions are presented as follows: [7] a) a lack of success at the end of the intervention; b) a high rate of participant dropout that induces low attrition, which influences the study’s power, the risk of bias and generalizability problems; [33, 37] c) the high economic costs associated with the demand for a large sample size; d) the risk of contamination of the control group, which may arise because of the initiation of healthy lifestyles in the absence of any specific intervention; and e) a lack of funding to support follow-up studies. To increase the number of follow-up studies after an intervention’s cessation, it is important to try to solve each of these factors to a) implement the interventions that report success; b) persuade the participants of the control group to continue in the study, for example, by offering to the control group the implementation of the intervention in their schools when a study demonstrates its effectiveness, and to motivate participants, it is important to involve them in the study process; c) simplify the analyzed variables to reduce the economic costs at follow-up; d) request that schools and participants if they receive any healthy lifestyle recommendation input to be aware about possible contamination; and e) plan the sustainability of the program before its implementation to assure its long-term funding [38].

Several limitations to our study were identified. First, despite the acceptable retention rates between the 2-year follow-up and the 4-year follow-up post-interventions, substantial dropout rates at the 2-year follow-up post-intervention were observed [18]. The low retention rates at the 2-year follow-up post-intervention that were observed in this study may be explained by the emigration phenomenon that occurred in Spain in 2013 in which approximately 30,000 children between ages 10 and 16 years emigrated with their parents [39]. In addition, the mobility of the participants from primary school to high school represented a considerable challenge in locating the adolescents at high school institutions. Moreover, the low retention rates may partially be due to the high rate of the parental decision to not provide consent and the rate of adolescents who themselves decided to not participate in the program. Also, the authors decision to exclude the adolescents birth before 01/01/1999 and after 31/12/2000 at 2-y follow-up post-intervention, could impact the final results. This decision was based on the participants birth between these dates presented the most sets of complete data of inclusion criteria [18]. Similarly, most other post-intervention follow-up studies have described high dropout rates as a major limitation [9, 12, 14]. However, the retention rate of the present study was higher than 25%, which may mean that the dropout rate did not significantly affect our results [40]. Moreover, dropouts were not differentially prevalent among the OW or OB adolescents and immigrant origin, which reduces the risk of bias [34]. However, a more through collection of demographic variables should be conducted in future studies. Second, there was an age difference between the intervention and control groups because most of the individuals in one group were born at the start of the year, and most of the individuals in the other group were born at the end of the year. Moreover, this study has not analyzed puberty, and this variable could act as a confounder. Third, there was a sample size ratio of 2:1 in favor of the intervention groups, which requires that our results be interpreted with caution.

Regarding generalizability, the original EdAl program is an easy-to-apply intervention that can be implemented in the primary schools of Spain to prevent childhood OB and improve lifestyles, with effects that extend from childhood to adolescence.

## Conclusion

At the 4-year post-intervention follow-up of the EdAl program, compared with the control groups, girls had lower BMI z-scores and boys had lower OB prevalence from the intervention. The encouragement in after-school PA was long-lasting and maintained after the cessation of the intervention, whereas healthy food habits must be further reinforced in adolescents.

## Additional file

Additional file 1: STROBE Statement—checklist of observational study items of EdAl study. (PDF 149 kb)

## Abbreviations

APPLE: A Pilot Programme for Lifestyle and Exercise
BMI: Body Mass Index
CATCH: The Child and Adolescent Trial for Cardiovascular Health
CHOPPS: CHristchurch Obesity Prevention Programme in Schools Study
EdAl: Educació en Alimentació (Education in Nutrition)
GLM: General Linear Models
HPA: Health Promoting Agent
IOTF: International Obesity Task Force
mITT: Modified intention-to-treat
OB: Obesity
OECD: Organisation for Economic Co-operation and Development
OW: Overweight
PA: Physical Activity
SD: Standard Deviation
WHO: World Health Organization
